# Influence of Electrical Anisotropy on Apparent Resistivity Responses in Tunnel Advance Detection: A Three-Dimensional Forward Modeling Study

**DOI:** 10.3390/s26144653

**Published:** 2026-07-22

**Authors:** Qian Liu, Mingxin Yue, Chao Chen, Kun Yang

**Affiliations:** 1 School of Transportation Engineering, Nanjing Tech University, Nanjing 211816, China; qianliu@njtech.edu.cn; 2 State Key Laboratory of Intelligent Construction and Healthy Operation and Maintenance of Deep Underground Engineering, Xuzhou 221116, China; 3 College of Materials Science and Engineering, Taiyuan University of Technology, Taiyuan 030024, China; 4 College of Mechanical Engineering, Taiyuan University of Technology, Taiyuan 030024, China

**Keywords:** water-bearing faults, direct current method, forward modeling, electrode layout, electrical anisotropy, apparent resistivity, tunnel advance detection

## Abstract

Water-bearing faults pose critical hazards in tunnel excavation due to sudden water inrush, making reliable advance detection essential. This study presents a systematic forward modeling framework that integrates COMSOL Multiphysics and MATLAB to simulate three-dimensional direct current (DC) responses ahead of the tunnel face. A three-dimensional finite-element model was developed to investigate the influence of electrical anisotropy on apparent resistivity under controlled geological conditions. Electrical anisotropy of both surrounding rock and water-bearing faults is incorporated to evaluate its influence on apparent resistivity. Numerical experiments investigate the effects of surrounding-rock and fault anisotropy and reveal the mechanism behind hourglass-shaped low-resistivity anomalies. The results also reveal systematic biases when anisotropy is neglected, including forward-shifted anomaly positions, overestimated lateral extents, and more diffuse anomaly boundaries. When anisotropy is considered in both the surrounding rock and the fault, the simulated anomaly closely matches the preset fault location, demonstrating improved localization accuracy. The modeling results clarify the effects of key parameters on apparent resistivity responses and improve the interpretation of low-resistivity anomalies. They also provide a theoretical basis for enhancing the reliability of DC resistivity-based tunnel advance detection.

## 1. Introduction

As tunnel construction moves into deeper and more geologically complex regions, the likelihood of encountering unfavorable geological conditions during excavation increases significantly. Water-bearing faults and fractured zones are particularly hazardous because they can trigger sudden water inrush events [1,2,3] and threaten construction safety. Reliable advance detection of geological structures ahead of the tunnel face is therefore crucial for risk mitigation and safe tunnel construction.

To reduce construction risks, tunnel advance detection has become an essential component of modern tunnel engineering. Among the available techniques, geophysical methods are widely used because they are non-destructive, efficient, and capable of investigating geological conditions ahead of the tunnel face over a relatively large range [4]. Recent studies have demonstrated that integrated geophysical investigations provide valuable support for geological hazard assessment and engineering decision-making in underground excavations [5]. The DC resistivity method is particularly suitable for detecting water-bearing faults, water-rich fracture zones, and other conductive geological anomalies by measuring apparent resistivity variations caused by electrical conductivity contrasts [6,7]. Because groundwater generally reduces the resistivity of rock masses, the DC resistivity method has become an important tool for the early warning of water-related geological hazards in tunnel construction.

In recent years, a large amount of research on direct current method advance detection has mainly focused on observation data processing and inversion interpretation [8,9,10,11,12,13]. Researchers are enhancing anomaly imaging through better electrode layouts, data acquisition, and 2D/3D inversion algorithms. With the development of computer technology, constrained inversion, joint inversion, and intelligent optimization algorithms have been gradually introduced into the field of geophysical inversion, improving the interpretation accuracy under complex geological conditions [14]. At the same time, machine learning and artificial intelligence technologies have also begun to be applied to anomaly identification and result interpretation, providing new ideas for improving detection efficiency [15]. However, the inversion method is essentially an indirect solution, and its results are affected by the initial model, regularization parameters and constraints, and non-uniqueness problems are common [16,17]. Different underground structures may produce similar observational responses, and the same set of observational data may also correspond to multiple geological interpretation results. In addition, on-site detection requires specially arranged instruments and testing work, which increases the difficulty and time cost of construction organization to a certain extent.

Forward modeling provides a systematic approach that starts from predefined geological models and directly computes electric field responses. This methodology allows the influence of individual geological parameters to be examined under controlled conditions. Compared to inversion methods, it allows precise evaluation of the effects of surrounding-rock and fault properties, offering a framework for anomaly prediction in tunnel advance detection. With a reasonable numerical model, we can systematically analyze how geological conditions affect detection responses, relate anomaly characteristics to observations, and provide a theoretical basis for survey design and interpretation [18,19,20]. In recent years, numerical calculation techniques such as finite element method, finite difference method and finite volume method have been widely used in forward modeling research based on direct current (DC) system. The research content mainly involves the analysis of the influence of anomalous body position, burial depth, scale, resistivity contrast, electrode arrangement and other factors on the detection results. With the development of multi-physics simulation platforms such as COMSOL Multiphysics, electric field simulation capabilities under complex three-dimensional geological conditions have been significantly improved, providing new technical support for the study of tunnel advance detection mechanisms.

Although some research has achieved certain results in direct current forward modeling, most of the existing models are based on the assumption of isotropic media, that is, assuming that the underground medium has the same conductivity in all directions. However, in actual engineering environments, surrounding rocks and faults are often affected by bedding, joints, fissures and tectonic processes, and exhibit obvious electrical anisotropy characteristics. Especially in rock masses with developed fissures and water-bearing faults, the migration of groundwater along the dominant direction will further enhance the directional difference in conductivity [21,22,23,24]. Current flow paths and electric field distributions differ markedly between anisotropic and isotropic media. These differences can alter anomaly amplitude, spatial extent, and boundary definition, reducing the reliability of advance-detection interpretation.

However, research on the effects of electrical anisotropy in tunnel advance detection remains limited. Although three-dimensional anisotropic resistivity modeling has been applied to the advance detection of water-bearing faults, previous studies have mainly focused on improving prediction performance under anisotropic conditions [25]. The physical mechanisms by which different anisotropic media affect apparent resistivity responses and anomaly characteristics remain insufficiently discussed. In particular, the respective influences of surrounding-rock anisotropy and fault anisotropy on anomaly morphology and localization have not been clearly interpreted. Addressing these issues is essential for improving the physical understanding and engineering interpretation of DC resistivity-based tunnel advance detection.

To address this gap, this study constructs a three-dimensional forward modeling framework combining COMSOL Multiphysics and MATLAB, focusing on water-bearing faults ahead of the tunnel face. A 3D tunnel model is established, and electrical anisotropy parameters for surrounding rock and faults are introduced. Different medium combinations are simulated to analyze the apparent resistivity response under complex geological conditions. The study investigates how anisotropy modifies low-resistivity anomaly responses and quantifies the errors caused by the isotropic assumption. The objectives are to quantify the influence of surrounding-rock and fault anisotropy on resistivity responses, assess interpretation biases, and establish a theoretical framework for anisotropy-aware tunnel advance detection. The results help explain electric-field responses in anisotropic media and support the identification of water-bearing structures ahead of the tunnel face.

## 2. Materials and Methods

### 2.1. Overall Framework

**Figure 1:**
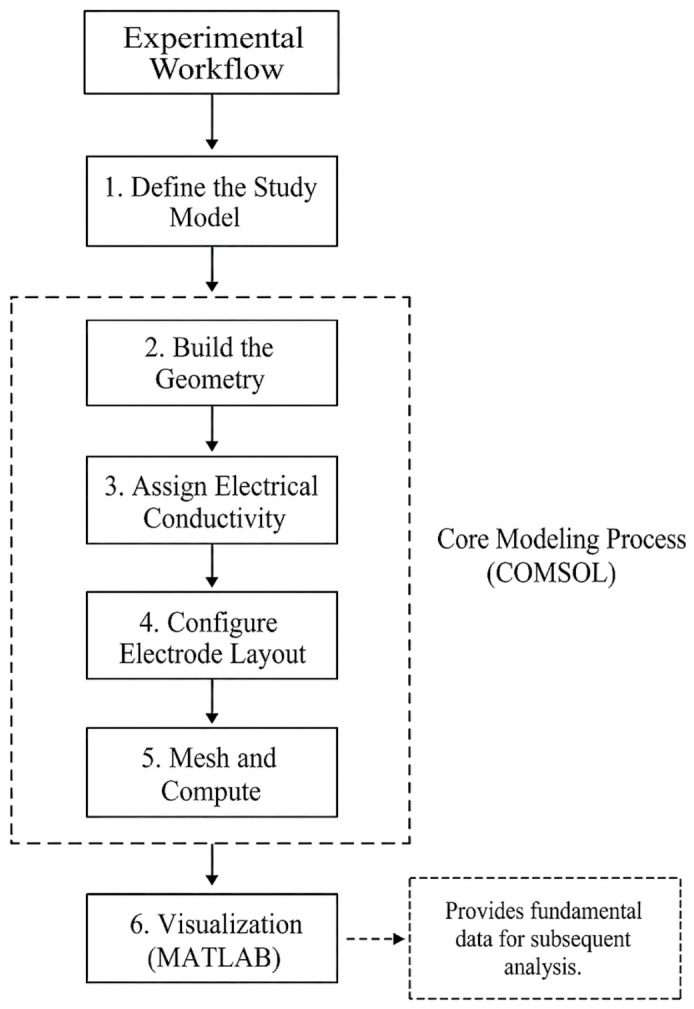
Overall workflow for forward modeling.

The experimental workflow is divided into six process-controlled steps: defining the research model, constructing the geometric model, assigning conductivity, arranging electrodes, meshing and calculation, and visualization. Steps involving geometric construction, conductivity assignment, electrode layout, meshing, and numerical computation are the core modeling processes conducted in COMSOL (version: 6.2), while MATLAB (version: R2024a) is used to visualize apparent resistivity distributions and compare the responses of different models. The flow chart of this experiment is shown in Figure 1.

### 2.2. Advanced Detection by DC Method

The DC resistivity method is a geophysical method that uses the resistivity difference of underground media to detect the spatial distribution of geological bodies. The basic idea is to supply a stable current to the underground medium, measure the potential difference generated in the surface or underground space, and invert the underground resistivity structure to identify geological bodies of different properties. The DC method is efficient and sensitive to anomalies (especially water-bearing ones), making it widely used in tunnels.

Resistivity ρ is an important parameter indicating the conductivity of the medium. In physics, when a current passes vertically through unit length and unit cross-sectional area, the resistance presented is the resistivity of the material:

(1) $$\rho =R\frac{L}{S}$$

In the formula, ρ is the resistivity, the unit is Ω•m; R is the resistance, the unit is Ω; L is the length, the unit is m; S is the cross-sectional area, the unit is m^2^.

The reciprocal of resistivity is called conductivity, represented by σ:

(2) $$\sigma =\frac{1}{\rho }$$

Conductivity is expressed in s/m. High resistivity (or low conductivity) indicates poor conductivity, and vice versa.

In a homogeneous isotropic medium, when a current electrode injects a steady current I into the ground, steady current and potential fields are established in the medium. According to the electric field theory, the potential generated by a point source in a semi-infinite homogeneous medium satisfies:

(3) $$V=\frac{\rho I}{2\pi r}$$

where V is the potential at a distance r from the current source, ρ is the resistivity of the medium, I is the intensity of the injected current, and r is the distance between the observation point and the current source.

However, natural rock masses often have significant heterogeneity and anisotropy. The resistivity of rocks is mainly controlled by factors such as lithological composition, pore structure, fracture development, moisture content, and groundwater salinity. When the current encounters a geological body with a resistivity difference from the surrounding rock, the current flow path is redistributed, resulting in distortion of the original electric field. Geological bodies including water-rich faults, fracture zones, clay interlayers, and water-filled karsts display low resistivity, whereas intact dense rocks, dry surrounding formations, and air-filled cavities exhibit high resistivity. This contrast underpins the use of the DC method for detection.

Different from the traditional surface resistivity method, tunnel advanced detection is a near-field technique implemented in a limited underground space. During the detection process, power supply electrodes and measuring electrodes are usually arranged on the tunnel face or tunnel side walls. By injecting current into the surrounding rock, an artificial electric field is established in the rock mass in front of the tunnel face. Due to the short distance between the electrode and the target area, the current can propagate directly in front of the tunnel face, so it has a high response sensitivity to geological anomalies in the excavation direction.

When an anomaly body exists ahead of the tunnel face, its resistivity contrast with the surrounding rock alters the current field distribution. For low-resistivity anomalies, current tends to converge toward the interior of the anomaly, forming a current concentration phenomenon. This effect causes changes in the potential field distribution in the observation area, which will appear as anomaly responses in the measurement data. By analyzing these electric field distortion characteristics, the location, scale, and electrical characteristics of the anomaly in front of the tunnel face can be inferred.

In order to quantitatively characterize the electrical properties of underground media, it is necessary to calculate the apparent resistivity based on the measured potential difference. Its expression is:

(4) $$\rho_{a}=K\frac{\Delta V}{I}$$

where $\rho_{a}$ is the apparent resistivity (unit: Ω•m), K is the device coefficient determined by the spatial position of the electrodes, ΔV is the potential difference between the measurement electrodes, and I is the supply current.

The apparent resistivity [26] is not the true resistivity of the underground medium, but the equivalent resistivity parameter inverted from the measured electric field response under the assumption of uniform underground conditions. When complex geological structures exist underground, the apparent resistivity reflects the comprehensive response of the electrical structure within a certain detection range.

In summary, the essence of advance detection of tunnels using the direct current method is to establish an artificial electric field near the tunnel face, and utilize the electric field disturbance effect caused by the resistivity difference between the anomalous geological body ahead and the surrounding rock. Through potential measurements and apparent resistivity calculations, this method enables early identification and localization of unfavorable geological bodies (e.g., fault fracture zones and water-rich areas), providing a reliable basis for safe tunnel construction.

### 2.3. Principle of Forward Modeling

DC forward modeling computes current and potential field distributions from known subsurface electrical structures and survey parameters, thereby obtaining the corresponding electrical responses. For steady-state electric field conditions, the current distribution in the underground medium satisfies the law of charge conservation and Ohm’s law, and its governing equation is:

(5) ∇•(σ∇V)=0

where V is the potential and σ is the medium conductivity. This equation describes the propagation law of electric current in heterogeneous media and is the theoretical basis for forward calculation of DC method.

Since the tunnel advance detection involves complex geometric structures such as the tunnel face, tunnel wall space, and front anomaly bodies, it is difficult to obtain analytical solutions to the control equations. Therefore, the COMSOL Multiphysics platform is used to perform numerical solutions based on the finite element method. First, we build a 3D geoelectric model that includes surrounding rock, tunnel cavity, and anomalies, assigning conductivity parameters to each domain. Then the positions of the power supply electrode and the measuring electrodes are set in the model, and the on-site electrification process is simulated through the current source boundary conditions. At the same time, the outer boundary of the computational domain is implemented using a far-field (infinite-element) boundary condition in COMSOL Multiphysics to simulate an unbounded geological space. No insulating boundary condition was applied in the final model.

Upon completing grid discretization, COMSOL applies the finite element method to solve the governing equations and generate the potential field distribution across the model. The potential differences and apparent resistivity responses are then computed from electrode measurements, enabling analysis of the anomaly’s location, extent, and electrical properties. This workflow links geological structures with their electrical responses and provides a basis for interpreting anomalies in tunnel advance detection.

### 2.4. Selection of Electrode Device

According to existing research [27,28,29], the tunnel direct current method for advanced detection mostly uses a pole–dipole array. The poe–dipole array provides a large detection depth and can obtain strong anomaly responses in a limited observation space. Compared with the symmetrical device, this device can effectively enhance the disturbance of the electric field distribution by the anomalous body in front, and improve the ability to identify low-resistivity targets such as water-bearing faults and water-rich fracture zones [30,31,32,33,34]. At the same time, its electrode layout method is simple, requiring only a small number of electrodes to be laid along the line near the tunnel face. It has the advantages of small construction interference, high detection efficiency, and strong engineering adaptability. For tunnel environments limited by space conditions, the pole–dipole array can balance both detection distance and resolution capabilities, and is one of the most widely used observation methods in direct current advance detection.

**Figure 2:**
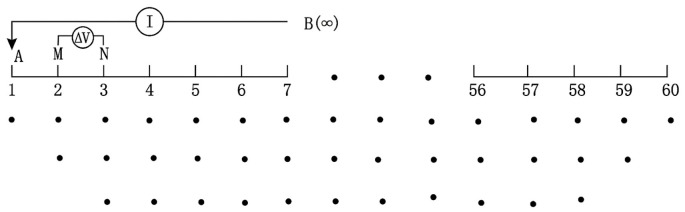
Diagram of pole–dipole array.

The pole–dipole array consists of a current electrode A placed along the survey line, potential electrodes M and N, and a return electrode B placed at infinity in the direction perpendicular to the survey line. The measurement process is shown in Figure 2:

The expression for the apparent resistivity of a pole–dipole array is:

(6) $$\rho_{s}^{AMN}=K_{AMN}\frac{U_{MN}}{I}$$

(7) $$K_{AMN}=2\pi \frac{AM•AN}{MN}$$

In the formula, $\rho_{s}^{AMN}$ is the apparent resistivity, K_AMN_ is the three-pole device coefficient, U_MN_ is the potential difference, AM, AN, and MN are the distances between the corresponding power supply electrodes and the measurement electrodes. In this configuration, the midpoint of the three-pole device MN is the recording point of the observation results. Equations (6) and (7) are derived from the electric potential of a point current source in a three-dimensional semi-infinite conductive medium and correspond to the standard apparent resistivity formulation for the pole–dipole array.

**Figure 3:**
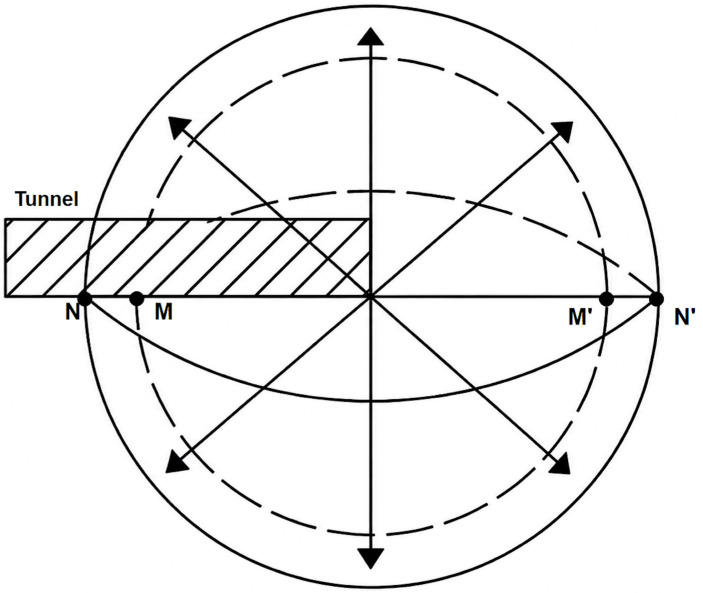
The principle of advance detection for pole–dipole array.

In 3D space, the pole–dipole array uses electrode A as the current source, while the other current electrode B is placed at a relatively infinite distance (or omitted). The potential difference between M and N is recorded to sense changes in subsurface electrical properties. With A as the center, a spherical equipotential surface is formed around point A (Figure 3). The change of the equipotential surface represents the comprehensive reflection of the electrical anomalies in the entire spherical shell. This is also the basic theory of the DC method for advanced detection.

### 2.5. Forward Modeling Process

This experiment establishes an experimental group and three control groups to analyze the impact of anisotropy on apparent resistivity. Among them, the surrounding rock and water-bearing faults in the experimental group are anisotropic; the surrounding rock and water-bearing faults in control group 1 are isotropic; the surrounding rock in control group 2 is anisotropic, and the water-bearing faults are isotropic; the surrounding rock in control group 3 is isotropic, and the water-bearing faults are anisotropic.

Model definition: Before formal 3D modeling, the basic configuration of the model needs to be clarified. In the research on tunnel advance detection, the elements of concern are: tunnel surrounding rock, tunnel cavity, and water-bearing faults. The surrounding rock area should be large enough to be considered as a semi-infinite medium. The fault is positioned at a realistic distance ahead of the tunnel face to ensure detectable but non-trivial electrical responses. Additionally, the entire model should be located at a certain depth underground.

Constructing a geometric model: In order to obtain reasonable results, the geometric parameters of this model are set as close to reality as possible. The surrounding rock of the tunnel is represented by a cuboid with a length of 300 m, a width of 200 m, and a height of 100 m; the tunnel is represented by a cylinder with a radius of 4 m and a length of 150 m (placed along the *x*-axis direction); the water-bearing fault is also represented by a small cuboid, 15 m long, 15 m wide, and 10 m high. In order to study the changes in apparent resistivity when the geological body is at different distances from the tunnel face, the fault position is changed sequentially and the calculation is performed. The water-bearing faults are located at 4 m, 8 m, and 12 m in front of the tunnel face. The selected fault distances (4 m, 8 m, and 12 m) represent near-, intermediate-, and relatively far-field positions within the effective investigation range of tunnel advance DC surveys. These representative cases allow the influence of target distance on apparent resistivity responses to be evaluated while maintaining a controlled and systematic comparison.

Setting conductivity: Surrounding rocks and faults are often controlled by bedding, joints, or fissures. Their electrical properties change little within the structural plane, but vary significantly in the direction perpendicular to the structural plane, so they can be approximately regarded as a transversely isotropic medium. When coordinate axes align with the principal conductive directions, the conductivity tensor simplifies to diagonal form, with equal resistivities in the x and y directions, leaving only horizontal-vertical anisotropy. The surrounding rock is assigned an intermediate conductivity representative of moderately conductive host rock. When electrical anisotropy of the surrounding rock is considered, the assigned conductivities are as follows:

(8) $$\left(\begin{array}{ccc} \sigma_{x} & 0 & 0\\ 0 & \sigma_{y} & 0\\ 0 & 0 & \sigma_{z} \end{array}\right)=\left(\begin{array}{ccc} 0.01 & 0 & 0\\ 0 & 0.01 & 0\\ 0 & 0 & 0.002 \end{array}\right)$$

where $\sigma_{x}$, $\sigma_{y}$, and $\sigma_{z}$ are the conductivities in the x, y, and z directions respectively, and the unit is: s/m.

If the surrounding rock is regarded as an isotropic material, the electrical conductivity is 0.01 s/m. The electrical conductivity of water-bearing faults is usually very high. When considering the anisotropy of water-bearing faults, the assigned values are as follows:

(9) $$\left(\begin{array}{ccc} \sigma_{x} & 0 & 0\\ 0 & \sigma_{y} & 0\\ 0 & 0 & \sigma_{z} \end{array}\right)=\left(\begin{array}{ccc} 0.1 & 0 & 0\\ 0 & 0.1 & 0\\ 0 & 0 & 0.02 \end{array}\right)$$

where $\sigma_{x}$, $\sigma_{y}$, and $\sigma_{z}$ are the conductivities in the x, y, and z directions respectively, and the unit is: s/m.

If the water-bearing fault is regarded as an isotropic material, the electrical conductivity is taken to be 0.1 s/m. A cylindrical tunnel is essentially a cavity and can be regarded as an isotropic material. Since the conductivity of air is very low, the conductivity of the tunnel is taken to be 10^−6^ s/m in this study. It should be noted that all parameter values are selected to represent realistic geological conditions. An anisotropy ratio of 5 between horizontal and vertical conductivity is adopted to enhance the general applicability of the results. The selected anisotropy ratio (5:1) is based on typical ranges reported in previous laboratory measurements and numerical studies of fractured rock masses [34], where anisotropy ratios commonly vary between 2 and 10 depending on fracture density and fluid content. This moderate value lies within a reasonable range for geological materials and allows anisotropic effects to be clearly identified without introducing extreme conductivity contrasts that may reduce the representativeness of the model.

The anisotropic conductivity values were selected to represent the directional electrical properties of layered surrounding rock commonly encountered in tunnel engineering, rather than to preserve an equivalent isotropic background conductivity. Therefore, the objective of the comparison is to evaluate the influence of realistic electrical anisotropy on apparent resistivity responses under representative geological conditions, rather than to perform a strict conductivity-controlled sensitivity analysis.

**Figure 4:**
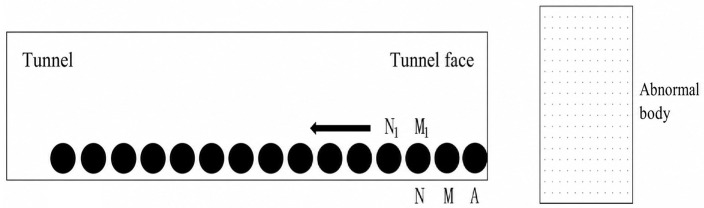
Layout of the pole–dipole array in the tunnel.

Laying out the electrodes: A pole–dipole array is used in this study. The power supply electrode A is arranged on the tunnel face, and the power supply current is 1 A. A current of 1 A is adopted because the governing equation of the DC resistivity method is linear under steady-state conditions. Although the electric potential varies proportionally with the injected current, the calculated apparent resistivity is normalized by the current intensity and is therefore independent of the selected current magnitude. Consequently, 1 A is used as a convenient reference current to simplify the numerical simulations and facilitate comparison among different models. Measuring electrodes are laid out sequentially along the side wall of the tunnel (opposite to the direction of tunnel excavation) with intervals of 1 m (Figure 4). The return current associated with electrode B is not explicitly modeled as a physical electrode within the computational domain. Instead, current conservation is satisfied implicitly in the infinite (open) domain, where the injected current disperses through the surrounding conductive medium and returns at infinity. This formulation is mathematically equivalent to a remote sink electrode and is commonly used in full-space resistivity modeling. A zero-potential reference (Dirichlet boundary condition, V = 0) is imposed on the far-field boundary to ensure a unique solution of the governing Poisson equation.

**Figure 5:**
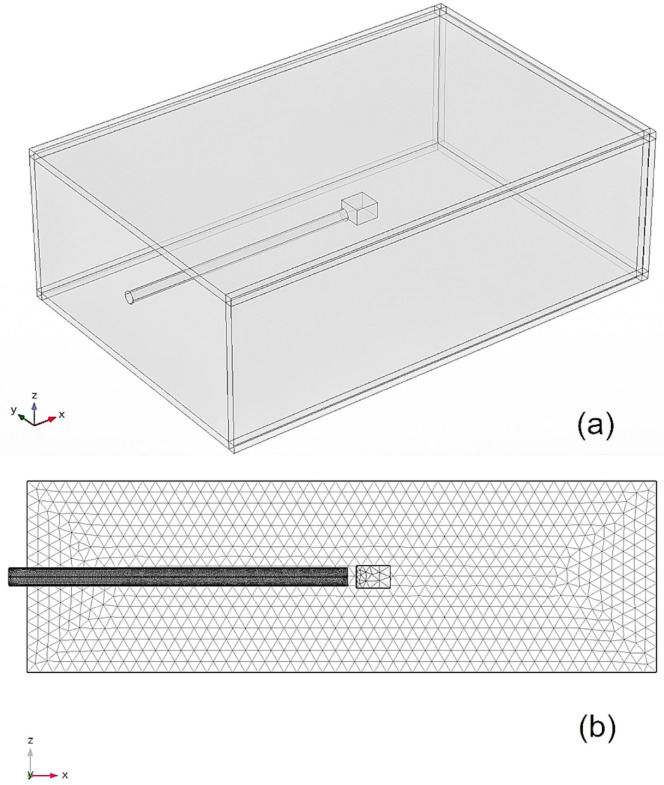
Diagram of the underground model. (**a**) Three-dimensional diagram of the overall structure. (**b**) Schematic diagram of the longitudinal section along the direction of tunnel excavation.

Meshing and calculation: After setting up the geometric model and material properties, the entire model is meshed. Steep potential gradients occur near the tunnel face, requiring fine mesh refinement to ensure accurate finite element solutions. Grid optimization enhances the fidelity of apparent resistivity calculations and reduces numerical error (maximum unit size: 6 m; minimum unit size: 0.6 m). The mesh is refined to 0.2 m near the electrodes and 3 m around the water-bearing fault to resolve steep potential gradients and conductivity contrasts. The final model consisted of 686,117 tetrahedral elements. Subsequently, a steady-state solver (a relative tolerance of 1 × 10^−3^) is used to calculate the finite element discrete model to obtain the potential distribution on the tunnel face and in the surrounding rock ahead. These potential distributions reflect the propagation characteristics of current in front of the tunnel, providing basic data for apparent resistivity calculation and anomalous body response analysis. The overall model is shown in Figure 5.

Visualization: We calculate apparent resistivity using Equations (6) and (7), then apply the ratio method for correction. Relevant code is written in MATLAB to plot the apparent resistivity profile ahead of the tunnel face. On the basis of visualization, geological interpretation is performed to verify the rationality of the model design.

## 3. Results

### 3.1. Ratio Method

The ratio method is an apparent resistivity processing technique that specifically corrects the impact of tunnel cavities, and is of great significance in tunnel advance detection [35,36]. This method uses mathematical transformation to eliminate the distortion effect of the high-resistance cavity in the tunnel on the electric field distribution, thereby obtaining a more realistic electrical response of the geological body. The core principle of the ratio method is based on the high-resistance characteristic of the tunnel cavity. Extremely high resistance significantly distorts the distribution of the current field. This distortion can lead to spurious apparent resistivity anomalies near the tunnel face, interfering with the accurate identification of water-bearing structures ahead. The ratio method converts the original apparent resistivity into the corrected apparent resistivity by introducing the correction coefficient c. The specific calculation formula is:

(10) $$\rho_{c}=\frac{\rho_{s}}{c}$$

The correction coefficient c is defined as the ratio of the apparent resistivity when there is no water-bearing anomaly to the background resistivity (in this experiment, it is also defined as the ratio of the apparent resistivity when there is no water-bearing fault to the resistivity of the surrounding rock). By removing the dominant background effect, the method enhances the visibility of weak anomalies associated with water-bearing faults. Compared with direct apparent resistivity interpretation, the ratio method provides a more stable representation of spatial variation patterns in complex tunnel environments. Therefore, the ratio method is mainly used as a data normalization technique to improve the interpretability of resistivity anomalies rather than to modify the physical forward model itself.

In this study, when the background surrounding rock is isotropic, the background resistivity can be taken as 100 Ω•m; when the background surrounding rock is anisotropic, the background resistivity is taken as the arithmetic square root of the transverse resistivity and the longitudinal resistivity, that is, 223.6 Ω•m.

Although no formal accuracy index was defined for the ratio-correction approach, comparison of the apparent resistivity images before and after correction shows that the tunnel cavity effect is effectively suppressed. This method removes the geometric effect of the tunnel cavity and better isolates the electrical response of geological anomalies, providing a reliable data basis for accurate prediction of water-bearing faults and fissure areas. The corrected apparent resistivity distributions provide a more reliable basis for geological interpretation.

### 3.2. Image Analysis

**Figure 6:**
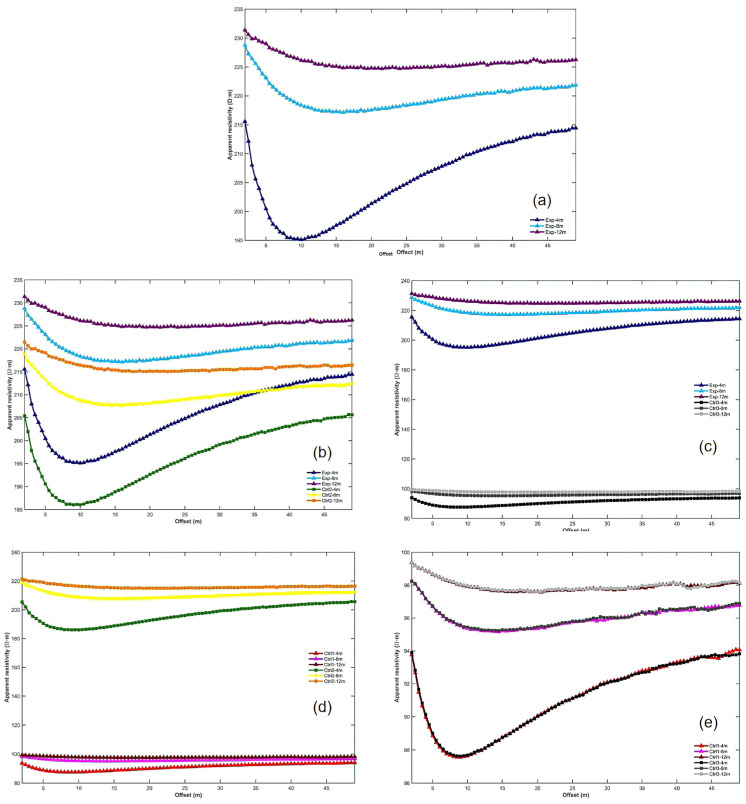
The apparent resistivity curve in front of the power supply electrode A.

The apparent resistivity diagram directly in front of the power supply electrode A (a line directly in front of A) is shown in Figure 6: panel (a) is the apparent resistivity curve of the experimental group; panel (b) is the apparent resistivity curve of the experimental group and control group 2; panel (c) is the apparent resistivity curve of the experimental group and control group 3; panel (d) is the apparent resistivity curve of control group 1 and control group 2; panel (e) is the apparent resistivity curve of control group 1 and control group 3.

The decrease in apparent resistivity indicates current concentration within the conductive fault. Apparent resistivity then recovers as the influence of the fault weakens with distance.

When the fault is 4 m or 8 m ahead of the tunnel face, the models show distinct low-resistivity anomalies: apparent resistivity decreases rapidly with distance from the tunnel face and then gradually increases after reaching the minimum. As the fault distance increases to 12 m, the anomaly amplitude weakens, indicating a reduced influence of the fault on the electric field.

Comparison with Control Group 2 shows that fault anisotropy mainly affects anomaly amplitude. The overall anomaly shape and position remain largely unchanged when surrounding-rock anisotropy is preserved. In contrast, altering the surrounding-rock anisotropy significantly changes both the curve level and the anomaly response, demonstrating that the surrounding rock exerts a stronger control on apparent resistivity.

Under the conductivity contrasts considered in this study, surrounding-rock anisotropy produces a larger perturbation in apparent resistivity than fault anisotropy. However, these results reflect only the local response along a single line directly ahead of electrode A. Their applicability must be evaluated together with the 2D apparent resistivity distributions.

**Figure 7:**
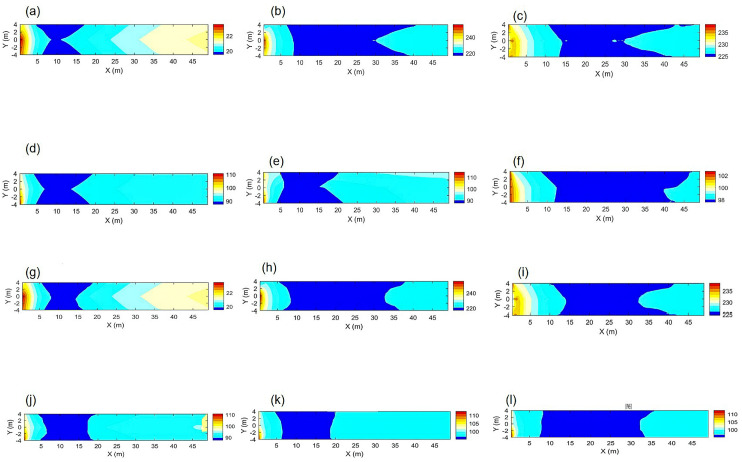
Apparent resistivity transverse section ahead of the tunnel face.

The apparent resistivity profile in front of the tunnel face is shown in Figure 7. Panels (a)~(c) are the cross-sections of the experimental group when the water-bearing fault is 4 m, 8 m, and 12 m away from the tunnel face respectively; panels (d)~(f) are the cross-sections of the control group 1; panels (g)~(i) are the cross-sections of the control group 2; panels (j)~(l) are the cross-sections of the control group 3.

As shown in the cross-sections of the experimental group, when anisotropy is considered in both the surrounding rock and the water-bearing fault, the low-resistivity anomalies first appear at approximately 4 m, 8 m, and 12 m, closely matching the preset fault locations. The difference between the anomaly onset position and the preset fault location is less than approximately 0.2 m, indicating high localization accuracy. In panels (a)–(c), the anomalies first appear at approximately 4 m, 8 m, and 12 m, matching the model settings. The low-resistivity anomaly areas also tend to narrow in the middle to a certain extent, making the entire low-resistivity anomaly area appear “hourglass-shaped”. The hourglass-shaped anomaly does not represent the actual fault geometry. Instead, it results from current redistribution caused by the electrode configuration and electrical anisotropy. Since the horizontal conductivity of the surrounding rock is significantly higher than the vertical conductivity, the current preferentially propagates in the horizontal direction and forms a convergence effect in the central area of the low-resistivity layer; at the same time, current diffusion occurs near the upper and lower boundaries of the anomaly, causing the low resistance response to extend to both sides. Ultimately, the apparent resistivity anomaly appears as an hourglass-shaped feature that contracts in the center and expands upward and downward. As the fault moves farther from the tunnel face, the spatial influence range of low-resistivity anomalies expands. This phenomenon does not represent an increase in the size of the anomaly, but is caused by an increase in the perturbation range of the low-resistivity layer to the surrounding current field. In anisotropic media, current preferentially follows high-conductivity directions, causing the anomalous response to spread farther forward and expanding the low-resistivity area. The spatial extent of the low-resistivity anomaly represents the range of electric field disturbance rather than the actual geometric boundary of the fault.

**Figure 8:**
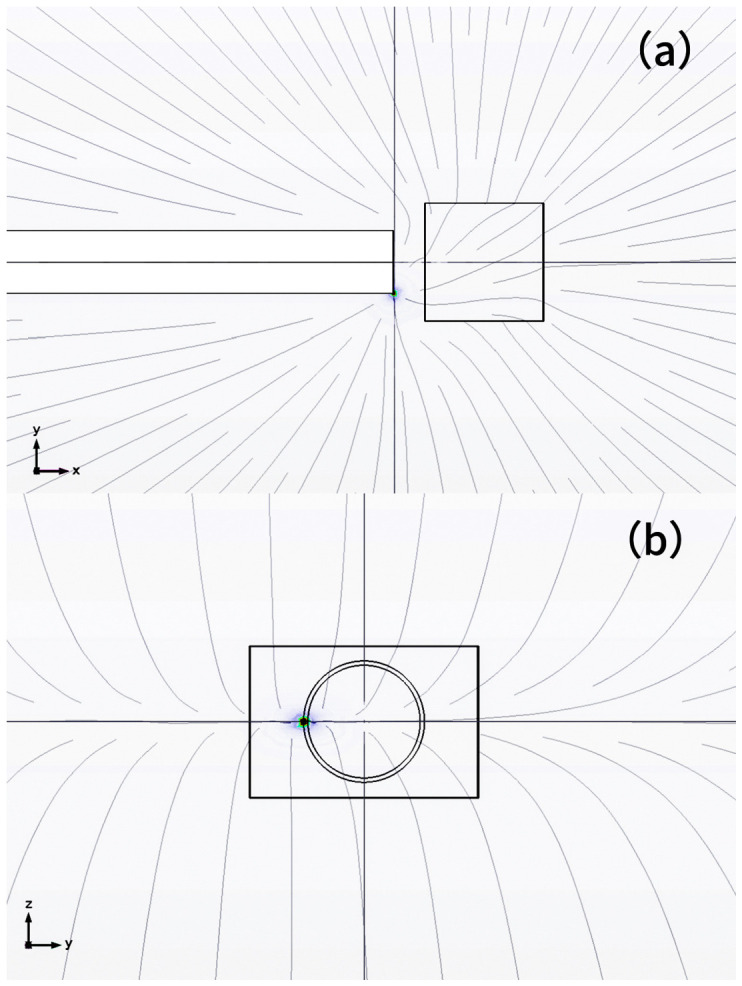
Distribution map of electric field. (**a**) Cross-sectional view of electric field distribution. (**b**) Longitudinal profile of electric field distribution.

The electric-field distribution (Figure 8) indicates that the fault modifies the propagation path of the electrical field rather than producing a response that directly reflects its geometry. Near the center of the fault, the field lines remain relatively concentrated, whereas noticeable divergence occurs near the upper and lower boundaries. This difference in perturbation range results in a narrower anomaly core and a broader response at the margins. Consequently, the apparent resistivity anomaly exhibits an hourglass-like shape, which reflects electric-field redistribution.

After the surrounding rocks and faults are simplified to isotropic media, the current propagates forward without directional constraints, and the disturbance of the low resistivity fault to the current field can be transferred to the area near the tunnel face earlier. Compared with the experimental group, Control Group 1 exhibits low-resistivity anomalies that appear more than 1 m closer to the tunnel face for the 8 m and 12 m fault cases, indicating that neglecting anisotropy causes a forward shift in the interpreted anomaly position and consequently underestimates the actual distance between the fault and the tunnel face.

In Control Group 2, the anomaly position and overall morphology remain similar to those of the experimental group, with only a slight increase in the lateral extent of the low-resistivity anomaly. This suggests that, under the conductivity conditions considered in this study, fault anisotropy has only a limited influence on the apparent resistivity response. In contrast, Control Group 3 exhibits a noticeable forward shift in the low-resistivity anomaly, indicating that surrounding-rock anisotropy plays a dominant role in controlling anomaly localization. Although the anomaly core becomes slightly more concentrated, the isotropic models generally produce a broader conductive response than the preset fault geometry. The prominent deep-blue low-resistivity zone in Figure 7 extends beyond the preset fault length of 15 m, indicating that neglecting anisotropy overestimates the apparent lateral extent of the conductive target. This enlarged anomaly reflects the spatial redistribution of the electric field rather than the actual geometric dimensions of the water-bearing fault.

The quantitative comparison indicates that, when both the surrounding rock and the fault are treated as anisotropic, the low-resistivity anomaly is located within 0.2 m of the preset fault position. In contrast, the anomaly displacement exceeds 1 m in the remaining comparison cases. These results demonstrate that incorporating anisotropy in both the surrounding rock and the fault significantly improves the localization accuracy of the anomaly.

Our forward modeling framework systematically reproduces expected electrical responses under various anisotropic conditions. Controlled comparisons among the experimental group and the three control groups highlight how surrounding-rock anisotropy dominates anomaly morphology and positioning. The numerical results reproduce the expected responses of water-bearing faults under different anisotropic conditions and show how anisotropy affects anomaly position and morphology. The simulated anomalies are generally consistent with the predefined geological models. More importantly, the results highlight the dominant influence of surrounding-rock anisotropy on apparent resistivity distributions. These results help explain the apparent resistivity characteristics observed in anisotropic tunnel environments. Compared with the control groups, the anomaly morphology obtained by the experimental group is generally more consistent with the preset geological model, and the anomaly location is more accurately depicted. The results also indicate that electrical anisotropy, particularly of the surrounding rock, can significantly affect apparent resistivity responses and should be considered in complex geological settings. Therefore, it is prudent to account for anisotropy in such analyses. Overall, the proposed forward modeling framework successfully reproduces the expected electrical responses of water-bearing faults under different anisotropic conditions. The results provide theoretical guidance for anomaly interpretation and may support future survey design and risk assessment in tunnel engineering.

## 4. Discussion

### 4.1. Scientific Contributions and Novelty

The novelty and scientific value of this study are as follows:

1. Quantitative evaluation of anisotropic effects in three-dimensional tunnel advance detection: Unlike previous forward modeling studies that commonly assume isotropic media, this study incorporates electrical anisotropy of both surrounding rock and water-bearing faults into a three-dimensional tunnel environment and systematically evaluates their influence on apparent resistivity responses.
2. Identification of the dominant role of surrounding-rock anisotropy: Comparative numerical experiments reveal that surrounding-rock anisotropy exerts a substantially greater influence on anomaly morphology and position than fault anisotropy. This finding provides new insight into the interpretation of electrical anomalies in complex geological settings.
3. Explanation of the formation mechanism of hourglass-shaped anomalies: The study demonstrates that the characteristic hourglass-shaped low-resistivity pattern results from anisotropy-induced current redistribution rather than the actual geometry of the fault. This provides a new physical interpretation of apparent resistivity anomalies in tunnel advance detection.
4. Establishment of a theoretical framework for anisotropy-aware interpretation: The proposed forward modeling framework clarifies the systematic interpretation bias introduced by the isotropic assumption and provides theoretical constraints for future inversion studies and field investigations. In addition to providing a qualitative interpretation of anisotropy-induced apparent resistivity responses, this study establishes a coupled COMSOL–MATLAB workflow for three-dimensional DC forward modeling in tunnel advance detection. Although both platforms are widely used individually, their integration provides a practical and reproducible technical route for model construction, numerical simulation, apparent resistivity calculation, and result visualization, which may facilitate future research on tunnel advance detection.

### 4.2. Analysis of Anisotropy Mechanism

Research results show that the controlling effect of surrounding rock anisotropy on electric field distribution is stronger than that of fault anisotropy. The hourglass-shaped anomaly does not reflect the fault geometry but arises because the surrounding rock’s horizontal conductivity exceeds its vertical conductivity, causing current to converge at the low-resistivity center and spread at the boundaries. This finding highlights a limitation of the isotropic assumption. Ignoring anisotropy shifts anomalies toward the tunnel face and underestimates the actual target distance. In engineering practice, this deviation may lead to misjudgment of construction risks or unnecessary advance support expenditures.

### 4.3. Engineering Implications

The findings demonstrate that incorporating electrical anisotropy into tunnel advance detection enhances the accuracy and reliability of anomaly identification. By accounting for surrounding-rock anisotropy, the proposed forward modeling framework can more precisely locate water-bearing faults, characterize their spatial extent, and predict the distribution of low-resistivity zones. This capability helps estimate the position and extent of conductive anomalies ahead of the tunnel face. The proposed model can be used to evaluate the influence of anisotropy on apparent resistivity responses and may assist the interpretation of field observations in tunnel engineering.

### 4.4. Limitations and Prospects

The geological model used in this study is an idealized representation of a tunnel environment. It was designed to isolate the influence of electrical anisotropy on DC resistivity responses under controlled conditions. This simplification reduces the coupling effects of multiple geological factors and improves the clarity of the physical interpretation. Although simplified, the model preserves the essential electrical contrast between the surrounding rock, fault zones, and tunnel cavity, which is the primary factor controlling apparent resistivity responses. Therefore, the present study focuses on revealing the underlying physical mechanisms rather than reproducing a specific engineering case, which is consistent with the objective of forward modeling analysis. Nevertheless, the conclusions should be interpreted within the scope of the adopted model. In practical tunnel engineering, geological conditions are often much more complex and may involve variable tunnel geometries, dipping faults, heterogeneous fault zones, irregular fracture networks, and spatially varying electrical properties. These factors may influence current-flow paths and modify the amplitude, spatial extent, and geometry of apparent resistivity anomalies. However, the proposed forward modeling framework is not limited to the simplified model presented here. By adjusting the geometric configuration and conductivity tensors, it can be readily extended to more realistic geological scenarios. Future work will incorporate more complex geological settings and conduct systematic investigations of inclined faults, heterogeneous fault properties, and irregular tunnel geometries to further evaluate the robustness and engineering applicability of the proposed framework. Although this study provides meaningful insights into the influence of electrical anisotropy, several aspects still warrant further investigation.

1. Refinement of the geometric model: The current fault model is relatively simplified. In fact, the anisotropy of the fault is controlled by factors such as fault inclination and fracture development, and the anisotropy tensor is very complex. Future studies should incorporate more complex random fracture networks to better simulate tectonically developed zones.
2. Multi-field coupling: Water inrush is a process of hydromechanical coupling. Subsequent attempts can be made to couple the seepage field with the electric field to improve the prediction accuracy of the dynamic evolution of water-rich structures.
3. Combined with field data measurements: The forward model established in this study can be used as prior constraints for inversion calculations. By constructing an initial geoelectric model that considers anisotropy, the non-uniqueness of the inversion can be significantly reduced. In field applications, measured data can be compared with a library of forward responses under various conditions, enabling rapid qualitative anomaly identification and quantitative parameter verification. This provides a physical basis for interpreting data in complex environments.

Although the present forward modeling framework successfully reproduces the expected electrical responses under different anisotropic conditions, it has not yet been validated against field measurements or laboratory experiments. Therefore, the conclusions of this study should be interpreted primarily as a mechanism-oriented investigation rather than a validated engineering prediction model. Nevertheless, the governing equations, finite-element implementation, and electrical parameters adopted in this study are based on established physical principles and values commonly reported in previous studies, providing a physically reasonable basis for the numerical simulations.

It should also be noted that the apparent resistivity response may be influenced by other factors, such as fault thickness, electrode spacing, conductivity contrast, and fault dip angle. These parameters were intentionally kept constant in this study to isolate the effect of electrical anisotropy. Future studies will investigate their influence through systematic parametric analyses under more realistic geological conditions. Future work will focus on validating the proposed framework through field measurements, laboratory-scale physical experiments, and comparisons with measured resistivity data from tunnel engineering projects. Such validation will further evaluate the predictive capability of the model, improve parameter calibration, and enhance its applicability to practical tunnel advance detection under complex geological conditions.

### 4.5. Supplementary Instructions

The pole–dipole array used here provides large detection depth and sensitivity in confined tunnels, causes little construction interference, and adapts well to engineering requirements. In addition, although the model simplifies the surrounding rock to a transversely isotropic medium, this is consistent with the characteristics of actual rock masses controlled by bedding or fractures. By setting up three control groups for comparative analysis, the interference of other variables is further eliminated and the rigor of the relevant conclusions is ensured.

In the present study, only a representative anisotropy ratio of 5:1 was considered to isolate the physical influence of electrical anisotropy under controlled conditions. Although this value falls within the range commonly reported for fractured rock masses, different anisotropy ratios may affect the magnitude and spatial distribution of apparent resistivity anomalies. Therefore, the conclusions presented here should be interpreted as mechanism-oriented rather than exhaustive over all anisotropy conditions. Future work will perform controlled comparative analyses across a wider range of anisotropy ratios to evaluate the robustness and general applicability of the proposed forward modeling framework.

The comparison between different control groups is primarily based on the forward modeling outputs, including apparent resistivity curves and spatial distributions. Although no additional statistical metrics are introduced, the variations in anomaly amplitude, position, and spatial morphology can be directly observed from the numerical results. These variations show consistent patterns across both 1D response curves and 2D cross-sectional images, which supports a reliable qualitative-to-semi-quantitative comparison. Since the study focuses on mechanism-oriented forward modeling, the visual and physical interpretation of numerical results is considered sufficient to distinguish the effects among different scenarios. Therefore, the conclusions drawn from the comparison are consistently supported by multiple independent model outputs.

## 5. Conclusions

This study investigates the influence of electrical anisotropy on three-dimensional tunnel advance detection using a DC resistivity forward modeling framework. A COMSOL–MATLAB coupled workflow is developed to simulate apparent resistivity responses of water-bearing faults under different anisotropic conditions. The results demonstrate that surrounding-rock anisotropy plays a dominant role in controlling electric-field redistribution and anomaly morphology, leading to a characteristic hourglass-shaped low-resistivity response. Quantitatively, when both surrounding rock and fault anisotropy are considered, the predicted anomaly location agrees closely with the preset fault position, with a localization error of less than approximately 0.2 m. In contrast, neglecting anisotropy results in a significant forward shift in the anomaly exceeding 1 m and an overestimation of the conductive zone extent. These results indicate that electrical anisotropy has a critical impact on the accuracy of anomaly interpretation in tunnel advance detection. The ratio-based processing method is used only to reduce tunnel cavity effects and enhance data interpretability. Overall, the study clarifies the physical mechanism of anisotropy-induced responses and provides quantitative evidence supporting the necessity of considering anisotropy in DC resistivity-based tunnel detection.

## Data Availability

The original contributions presented in this study are included in the article. Further inquiries can be directed to the corresponding author.

## Abbreviations

The following abbreviations are used in this manuscript:

DC	Direct current
3D	Three-Dimensional
